# Effect of Health Messages on Alcohol Attitudes and Intentions in a Sample of 16–17-Year-Old Underage Drinkers

**DOI:** 10.3390/ijerph14101183

**Published:** 2017-10-05

**Authors:** Antony C. Moss, Stephen Evans, Ian P. Albery

**Affiliations:** School of Applied Sciences, London South Bank University, London SE1 0AA, UK; evans653@btinternet.com (S.E.); alberyip@lsbu.ac.uk (I.P.A.)

**Keywords:** alcohol, attitude change, drinking intentions, student drinkers, underage drinkers, responsible drinking messages

## Abstract

*Background:* Responsible drinking messages (RDMs) are a key component of many education-based interventions for reducing alcohol harms. The evidence base for the effectiveness of RDMs is extremely limited, with some recent research suggesting iatrogenic effects of such messages. *Objective:* To examine the effects of exposure to health messages on attitudes towards drinking and drunkenness, and intentions to drink and get drunk, amongst underage drinkers. *Methods:* Ninety-four underage drinkers were recruited from colleges in the UK. Participants were either actively or passively exposed to one of two health messages (RDM or general wellbeing). Measures of attitudes and intentions towards drinking and drunkenness were obtained one week before and immediately after participation in the study. A unit estimation task was also included. *Results:* Active exposure to RDMs led to more positive attitude towards drunkenness, while passive exposure led to more negative attitudes. Passive RDM exposure led to increased intentions to get drunk in future. Wellbeing posters produced the opposite effect in some but not all of these measures. *Conclusions:* Exposure to RDMs may have some beneficial effects in terms of creating more negative attitudes towards alcohol consumption, but we also identified potential iatrogenic effects regarding attitudes and intentions towards drunkenness amongst an underage sample of drinkers. Further research is required to better understand optimal ways of framing RDMs to produce positive changes in attitudes, intentions, and prospective drinking behaviour.

## 1. Introduction

Underage drinking in the UK is generally considered to be drinking by anyone under the age of 18. While under limited circumstances it is legal for 16–17 year olds to consume alcohol (i.e., when an adult buys an alcoholic drink for a 16–17-year-old in a licensed premises, when consumed with a meal), the legal age for the purchase of alcohol is 18 years. While underage drinking has been on the decline for a number of years across the UK (e.g., [1]), those children who do drink in the UK tend to consume larger quantities of alcohol relative to other European countries [1,2], which represents an important public health challenge.

Health messages in the form of posters, leaflets, and television advertisements are widely used to improve knowledge and understanding amongst the general public. Responsible drinking messages (RDMs) aimed at encouraging consumers of alcohol to avoid harmful drinking behaviours are often disseminated through these kinds of mass media. Such messages might include the provision of advice regarding responsible drinking behaviours, such as eating food when drinking on a night out, or alternating between soft and alcoholic drinks throughout a drinking session. Although such messages are widely used, research evidence regarding their efficacy is severely lacking. Indeed, in a recent systematic review [3], we identified only eight empirical studies which directly evaluated the effectiveness of RDM campaigns. While findings from this limited body of work generally suggest that such messages can have a positive impact (e.g., [4,5]), there is also some evidence suggesting that these campaigns may have iatrogenic effects under certain conditions [6].

So, RDMs in the present context are specifically limited to messages which aim to promote alcohol consumption patterns which are likely to reduce—or indeed, entirely avoid—harm. Amongst adult populations, such messages might, for example, encourage consumers to avoid excessive pre-drinking before a night out, suggest reducing the volume of alcohol consumed on a given night out (e.g., through alternating alcoholic and non-alcoholic drinks, or drinking beverages which contain less alcohol), or promoting the health benefits of alcohol-free days and reducing one’s overall intake over a more extended time period. Therefore, RDMs are distinguished from other forms of information dissemination which either utilise fear appeals or which seek to educate consumers about the alcohol content of different drinks (e.g., container-side labelling which lists the number of units in a given drink).

Educational interventions targeting children and young people are another means by which harm from drugs, alcohol, and other health behaviours can be minimised across the lifespan. This is an area where a larger quantity of evaluation research exists. In general, findings suggest that small but significant effects of school-based interventions can be shown (see [7] for a review). In terms of the overall effect size, such interventions are not as impactful as other population-level measures which seek to restrict availability—for example through minimum unit pricing, increased taxation, and restrictions on alcohol outlet densities [8]. Despite this, it is reasonable to conclude that such interventions ought to be implemented as part of a multicomponent response to tackling alcohol-related harms at a societal level.

While some research has evaluated the effectiveness of RDM campaigns amongst adults, and a more extensive literature base exists evaluating the impact of preventive interventions targeting school children, there is an absence of any research which has examined the impact of population-level RDM campaigns on children. While such campaigns are not specifically designed to target this age group, the prominence of their display via public media (e.g., billboards and posters) means that underage drinkers will nonetheless be exposed to them. In the present study, we sought to evaluate the impact of an RDM poster campaign compared with a more general wellbeing (GWB) campaign (which encouraged a healthy lifestyle without specifically mentioning alcohol or drugs) in a sample of underage drinkers in the UK. In addition to assessing the impact of posters, we also sought to examine the effect of passive and active engagement with their contents. Given that poster and national media-based campaigns, by their very nature, cannot ensure (or even encourage) active engagement with the message contents, we were keen to explore whether any effects of exposure to posters were affected by explicit engagement in a discussion about their core message, as opposed to the more passive exposure which would typically be used for such campaigns.

## 2. Method

### 2.1. Participants

Ninety-four college students who reported drinking at least one alcoholic drink in the past month and who were also below the legal age for alcohol consumption in the UK took part in the study. In a UK educational context, college students are typically 16–18 years of age, completing tertiary educational qualifications prior to university. Colleges are also open to students returning to education later in life, though the majority of students are in the younger age range. Colleges are almost exclusively non-residential, and so students would typically be living at home and commuting to a local college.

Participants were recruited from five different colleges in the Greater London area. Participants were recruited on the basis of their drinking status, so non-drinkers were not eligible. This was assessed during the first session, when baseline measurements were obtained from students who expressed an interest in participation. Participants were all A-level students studying psychology as one of their course options. All participants were either 16 (*n* = 49) or 17 (*n* = 39) years old. The sample included 73 (83%) females and reflects the gender distribution of the population from which the sample was recruited. AUDIT-C scores ranged from 2–11 (*M* = 5.7, *SD* = 2.8). The AUDIT-C (see Section 2.3, below) is a brief measure of alcohol consumption, and is used here to assess drinking patterns.

The study received ethical approval from the University’s Local Research Ethics Committee. In addition, permission to recruit participants from the colleges was obtained from the heads of each institution.

### 2.2. Design

A 2 × 2 × 2 mixed factorial design was used with Poster Type (Responsible drinking message (RDM) vs. General Wellbeing (GWB)) and Exposure Condition (Passive vs. Active) as between-participants factors, and Time (Pre- vs. Post-exposure) as a within-participants factor. To avoid contamination effects, assignment to each condition was based on recruitment location, rather than randomly on a participant-by-participant basis; therefore, the study used a quasi-experimental design. AUDIT-C scores and location were included in the multivariate analyses as covariates.

### 2.3. Materials

**Posters**. Two sets of five posters were used for each condition. The RDM posters were taken from a national campaign called “*Why Let the Good Times Go Bad?*” (active at the time of the study—see [9] for sample campaign materials), and contained simple suggestions aimed at encouraging responsible drinking behaviour. The GWB posters were taken from another national campaign—also current at the time of the study—called “*Change 4 Life*” (see [10] for sample materials and the original campaign brief). This campaign provided various simple suggestions aimed at improving health. The posters were the same as those used by Moss et al. ([6], Experiment 3). Each of these campaigns were aimed at a national audience across the UK, and comprised both poster-based messaging with various other supporting media, including websites providing more detailed information related to the specific campaigns, and the health behaviour changes they promoted.

Exposure to these stimuli was either passive or active. Passive exposure to the stimuli involved posters being displayed on the walls at the front of the students’ classroom while being given a brief 15-min interactive lecture on research methods in psychology which encouraged group discussion. In the active exposure conditions, each of the five posters were presented to the class, and participants were encouraged to discuss what they felt was the key message from each poster. This discussion also lasted for 15-min, in order to parallel the passive exposure condition.

**AUDIT-C**. The AUDIT-C [11] questionnaire is a three-item measure of problem drinking which—for non-dependent populations—has been shown to perform comparably to the full 10-item AUDIT questionnaire. Questions assess frequency of drinking, quantity consumed per drinking session, and binge drinking frequency. Responses are scored from 0–4 for each question, generating a total scale range from 0–12, with higher scores representing an increased risk of problem drinking.

**Drinking Attitudes and Intentions Questionnaire**. Items from the questionnaire developed by Davies and Foxall [12] were used to assess attitudes towards *drinking alcohol* and *drunkenness*, as well as self-reported intentions to *drink alcohol* and *get drunk at the weekend* following completion of the questionnaire. Attitudes towards “Drinking alcohol” and “Getting drunk” were assessed using four seven-point semantic differential scales with the adjective pairs “*bad-good*”, “*foolish-wise*”, “*unhealthy-healthy*”, and “*unpleasant-pleasant*”. Intentions were assessed using a single seven-Point Likert scale (“Extremely Unlikely–Extremely Likely”) for the statements “*I will drink alcohol this weekend*” and “*I will get drunk this weekend*”.

**Pouring Estimation Task**. Participants were asked to pour a single measure or “shot” (25 mL, approx. 1 UK unit) from a vodka bottle in to a 200-mL drinking glass. The vodka bottle actually contained water due to the fact that the study took place in a college setting and that the students recruited were below the legal age (i.e., 18 years) for drinking in the UK. Pouring tasks of this kind have been used previously to assess the impact of environmental manipulations on amount poured [13], and also to assess knowledge of alcohol units [14]. While we have previously shown that the amount poured in such tests can vary as a function of individual differences (i.e., strength of habitual drinking, [13]), in the present study we included this measure to determine whether environmental manipulations—in the form of health messaging—might impact the volume poured by participants.

### 2.4. Procedure

The Heads of a number of colleges were contacted to gain permission to recruit students from their institutions. Once this initial approval was received, arrangements were then made regarding convenient times to visit the recruit participants from the student body. The students who expressed an interest in participating were told about the nature of the research and handed an information sheet which contained details of what was expected of them should they agree to take part in the research. Participants were told that the primary purpose of the study was to understand drinking patterns amongst underage drinkers.

Once students agreed to participate, they were asked to complete the AUDIT-C and drinking attitudes and intentions questionnaire on their next visit to their college. Once they had completed the questionnaire, the participants were informed of the time and date the following week of a second research phase. In this phase, participants were randomly allocated, on the basis of institution, to one of the four experimental conditions (active vs. passive exposure to the RDM posters by active vs. passive exposure to the GWB posters). In the active exposure conditions, the researcher engaged in a discussion with each group regarding their views and understanding of the posters for approximately 15–20 min. Each poster was displayed for the same length of time, and the role of the researcher was primarily to encourage participation from all students in the group in sharing their views. In the passive exposure conditions, the posters were displayed on the wall at the front of the class, while the researcher facilitated a discussion on the nature of conversations and social interaction. In all conditions, participants then completed the drinking attitudes and intentions questionnaire for the second time. To maintain the credibility of the priming manipulation used in this study (i.e., the display of posters and/or discussion session about the posters), participants were told that the researcher was facilitating this discussion as a reward for their participation in the questionnaire-based research project. This would not be considered unusual in a UK college context, where staff from universities often visit colleges to deliver talks as part of programs of outreach activity.

Once they had completed the questionnaire, participants were asked to approach a table in the classroom and were asked to estimate and pour a 25 mL measure (i.e., one UK unit of alcohol) from a 1.5 L bottle of peach schnapps (which in fact contained water) into a clear plastic cup. Participants performed this in isolation from one another to avoid biases in estimation accuracy. The researcher then took an accurate measure of the amount poured. All participations were shown the correct volume for one unit at the end of the study.

All participants were debriefed at the end of the study and the heads of each college were provided with guidance which could be circulated to students regarding support services available if they had concerns about their own or others’ alcohol use.

### 2.5. Approach to Analysis

**Attitude Scale Reliability.** To determine the reliability of the attitude measures used in this study, a series of Cronbach’s alpha tests were conducted on the four items comprising each attitude measure at both Time 1 and Time 2. Results showed good reliability for all four attitude measures at both Time 1 (α_drinking_ = 0.86, α_drunkenness_ = 0.87) and Time 2 (α_drinking_ = 0.85, α_drunkenness_ = 0.89). The results of these tests are shown in Table 1, and demonstrate good reliability for each of the scales. Subsequent analyses report the mean score of the four items for each attitude scale.

**Group Comparisons.** A one-way analysis of variance was used to test for difference in AUDIT-C scores across the four experimental groups. Results demonstrated that there was no significant difference in AUDIT-C scores across the four conditions, *F*(3, 87) = 0.80, *p* > 0.05.

A series of one-way ANOVAs were then conducted to test for baseline differences in each of the six attitude and intention measures (i.e., Time 1 measures of attitude towards alcohol and drunkenness, intentions to drink alcohol and get drunk at the weekend, and beliefs about frequency of drinking and getting drunk at 18 years of age). Results showed there were no baseline differences across any of these measures (*F*s < 2, *p*s > 0.05). As a result, change scores were calculated for each dependent variable using Time 1 and Time 2 responses. Scores above zero indicated a more positive attitude/stronger intention. Subsequent analyses utilized and reported these change scores.

**Correlation Analysis.** For all participants, across all conditions, there were significant positive correlations between AUDIT-C scores and all six of the baseline measures of attitude, intention, and beliefs about future drinking. Pearson correlations are shown in Table 1. These results show that heavier drinkers were more likely to express positive attitudes towards drinking alcohol and drunkenness, as well as having greater intentions to drink alcohol and get drunk at the weekend, and beliefs about their drinking and drunkenness when they are 18. As a result, AUDIT-C scores were included in subsequent analyses as a covariate to control for the effects of differences between drinker types.

**Data Screening**: Prior to calculating change scores for each dependent variable, data were screened for normality and outliers were removed (though in practice no outliers were identified). Box’s M was not significant, suggesting that within-group covariance was equal across cells in the MANOVA (Box’s M = 81.97, *p* > 0.05). In addition, Levene’s test was not significant for all dependent variables, confirming that the assumption of equality of variance was met (*F*s < 2, *p*s > 0.05).

**Multivariate Analysis:** To explore the effect of poster type and exposure condition on attitudes towards drinking and getting drunk, intentions to drink and get drunk, and beliefs about frequency of drinking and getting drunk when participants turned 18, a multivariate analysis of covariance (MANCOVA) was conducted with poster type (RDM vs. GWB) and exposure condition (Passive vs. Active) as between participants variables. AUDIT-C scores, recruitment location, and gender were included as covariates, with change scores for *attitude towards drinking alcohol*, *attitude towards getting drunk, intentions to drink at the weekend, intentions to get drunk at the weekend*, beliefs regarding *how frequently I will drink alcohol when I am 18* and *how frequently I will get drunk when I am 18* as the dependent variables. Table 2 shows the mean responses to each dependent variable at time 1 and 2, across all conditions. Results of the overall MANCOVA model are displayed in Table 3.

## 3. Results

### 3.1. Multivariate Analyses—Changes in Attitudes, Intentions, and Beliefs about Future Drinking

**Effect of the Covariates:** Results show a significant multivariate effect of AUDIT-C scores on the combined dependent variables. Univariate analyses of this effect reveal that this was due to a significant effect of AUDIT-C score on changes in attitude towards both drinking alcohol and getting drunk—heavier drinkers demonstrated more positive changes in these attitudes across all conditions at time 2. Neither gender nor recruitment location significantly affected the combined dependent variables, and there were no significant univariate effects of either covariate on the dependent variables.

**Effect of Poster Type:** The multivariate effect of poster type approached significance (*p* = 0.08), and examination of the univariate effects demonstrated a significant effect of poster type on changes in attitudes towards drinking alcohol—participants exposed to the GWB posters demonstrated improvements in attitudes (*M_change_* = 0.31, *SEM* = 0.15) compared to participants exposed to the RDM posters, who demonstrated a decrease in attitude score (*M_change_* = −0.19, *SEM* = 0.16). There were no univariate effects of poster type on intentions to drink and get drunk at the weekend, nor beliefs about frequency of drinking and getting drunk at 18.

**Effect of Exposure Condition**: The multivariate effect of exposure condition was not significant, with no significant univariate effects for any of the six dependent variables.

**Poster Type × Exposure Condition Interaction:** A significant multivariate effect for the poster type × exposure condition interaction term was identified. Examination of the univariate effects showed significant interaction effects for the change in attitudes towards drunkenness, and for beliefs about the frequency of getting drunk at 18.

**Figure 1:**
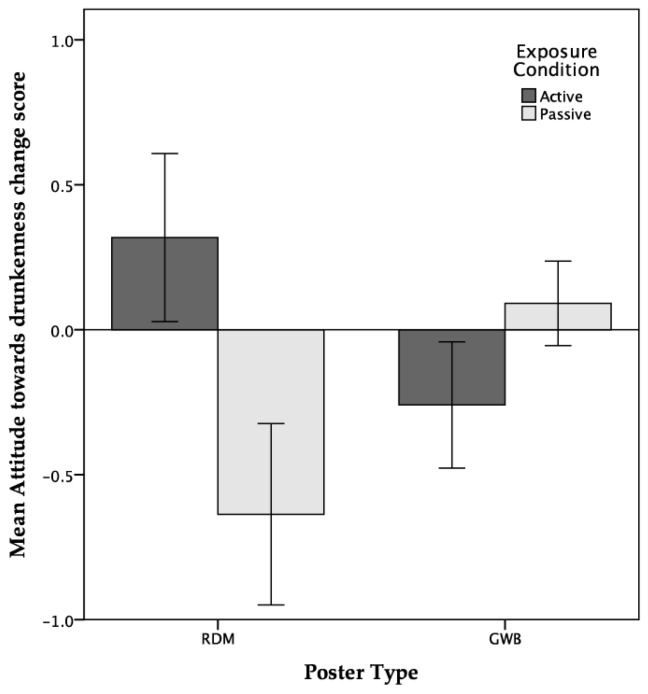
Interaction between poster type and exposure condition for attitudes towards drunkenness (error bars display ± 1 *SEM*).

To explore these interactions, simple main effects analyses were conducted using the Bonferroni correction. Results showed that passive exposure to RDM posters led to the development of significantly more negative attitudes towards drunkenness compared with both active exposure to RDMs and passive exposure to GWB posters (see Figure 1).

**Figure 2:**
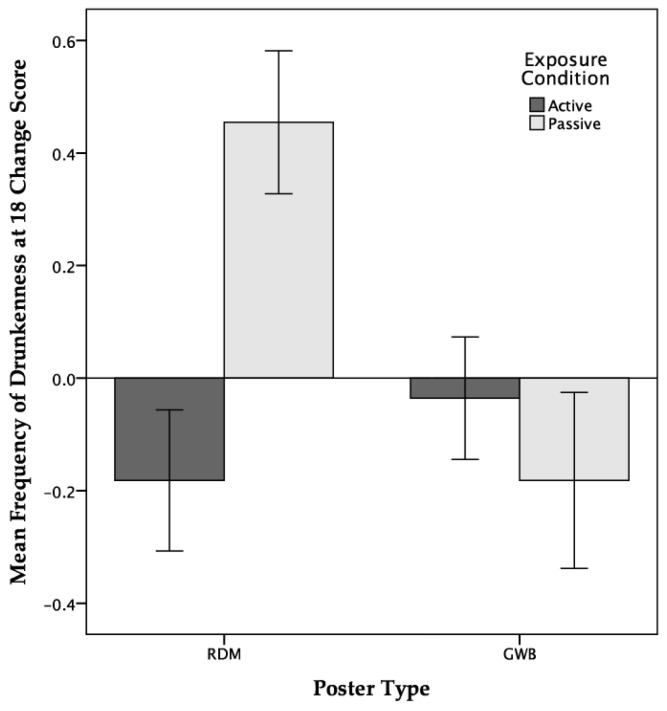
Interaction between poster type and exposure condition for beliefs regarding frequency of drunkenness at 18 (error bars display ± 1*SEM*).

With regards to beliefs about the frequency of getting drunk at 18, simple effects analysis revealed that participants who were passively exposed to the RDM posters reported significantly increased beliefs that they will get drunk at 18 compared with participants who were actively exposed to RDM posters and participants who were passively exposed to GWB posters. Both of these groups saw a reduction in their beliefs about their frequency of getting drunk at 18 (see Figure 2).

### 3.2. Pouring Estimation Task

A two-way ANOVA was conducted to determine whether there was an effect of poster type (RDM vs. GWB) and exposure condition (Passive vs. Active) on the amount poured (mL) during the pouring estimation task. On screening data for normality, six cases were identified as extreme outliers (+3SD above the mean) and so were excluded from the analysis. The results of the ANOVA revealed no significant main effects of poster type (*F*(1, 84) = 2.33, *p* = 0.13, *η_p_*^2^ = 0.03) or exposure condition (*F*(1, 84) = 0.10, *p* = 0.75, *η_p_*^2^ = 0.01) on amounts poured, nor was there a significant interaction (*F* = (1, 84) = 0.59, *p* = 0.44, *η_p_*^2^ = 0.07).

Across all conditions, the mean amount poured was 40 mL (SD = 11; range = 11–64), which is equivalent to 1.6 times the correct volume. Seventeen percent of participants poured the correct amount or less and 17% poured twice the correct amount (50 mL) or more. Allowing for a margin of error of 10% above or below the target amount (following [12]), only 19.3% of participants were able to correctly pour the target amount.

## 4. Discussion

We explored the effects of different types of health promotion messages, as well as different modes of delivery, on attitudes towards drinking and drunkenness, and intentions to drink and get drunk amongst a sample of 16–17 year old UK college students. An overall effect of RDM poster exposure was identified, with exposure to RDM posters leading to significantly more negative attitudes towards drinking alcohol compared to those exposed to GWB messages, who were shown to hold more positive attitudes towards drinking alcohol.

Neither exposure mode nor poster type had any significant effect on short-term intentions (i.e., the following weekend) to drink alcohol or get drunk at the weekend. However, when questioned about beliefs regarding the frequency with which they expected to be both drinking alcohol and getting drunk in the longer term (i.e., when participants turned 18, the legal age of alcohol consumption in the UK), a significant interaction between poster type and exposure condition was detected for expectations regarding drunkenness. Interestingly, the pattern of changes as a consequence of exposure to RDM posters and exposure condition was exactly opposite to those findings regarding changes in attitudes towards drunkenness. Specifically, participants actively exposed to RDM posters reported more positive attitudes towards drunkenness but a reduction in the frequency with which they expected to get drunk at 18. Conversely, participants who were passively exposed to RDM posters reported more negative attitudes towards drunkenness but an increase in the frequency with which they expected to get drunk at 18.

In the alcohol pouring task, although there were no significant effects of poster type or exposure condition on accuracy in judging the volume of one unit of alcohol, the majority of participants significantly misjudged the volume, with more than 80% overestimating (by 10% or more) the correct volume. This finding with an adolescent sample is consistent with findings from an adult sample in Scotland [15]. However, in terms of those who poured accurately, fewer participants in the present study were able to do this compared to Gill and O’May’s [12] sample (17% vs. 27%, respectively). This finding is of particular importance, given that the present sample comprising underage drinkers would be more likely to be drinking outside of licensed premises where drinks are usually served in standard measures. As a consequence, even if motivated to moderate their alcohol intake in line with unit guidelines for adults, they would be at increased risk of underestimating how much alcohol they are actually consuming.

The results from this study are somewhat equivocal as to the effects of RDM exposure. While passive exposure led to more negative attitudes towards drunkenness, active exposure had the opposite effect—and we also identified an increase in longer term intentions to get drunk at 18 when participants were passively exposed to RDMs. Previous research—albeit with older age groups (and, it is important to note, who were above the legal age to drink)—has shown a similar pattern of equivocal findings, with iatrogenic effects noted in one study [6], and positive effects in others [4,5]. Interestingly, our own study in which iatrogenic effects were noted utilized the same posters as the present study—which suggests that there may be a specific effect of the campaign being evaluated here, and that this effect may not generalize to RDMs in general. However, it is also important to consider that our own previous study differed from many other studies in the literature, in that we utilized a simulated bar environment to test the effects of the RDM campaign, while other studies utilized methods such as exposure to RDMs via web-based methods (e.g., [5]).

The present study has some limitations, notably the overrepresentation of female participants. The study sample was itself also relatively small, and so the absence of effects in some parts of the analysis may be due to low power. Future research exploring gender differences in attitudes towards alcohol and the impact of different forms of RDM would be worthwhile, as well as studies engaging larger samples across a wider range of settings. Indeed, it has been shown in previous research that a degree of message tailoring in the promotion of responsible drinking may be more efficacious than generic messages (e.g., [16]). It was also unfortunate that we were unable to obtain sufficient data regarding prospective drinking across all conditions (an attempt was made to collect prospective drinking data via an online drinking diary, but response rates were extremely poor), which naturally limits the conclusions which can be drawn with regards to the more causal impact of these different messages.

It is also important to note that our study did not include an active control group; rather, exposure to GWB and RDM messages were compared as a function of active or passive exposure to the message content. This limits the extent to which we are able to draw any conclusion regarding differences observed in relation to an active control receiving no information of any kind. Indeed, in one of our own previous studies, we observed that participants who were not exposed to any form of RDM (online information or RDM posters in a bar setting) drank the least amount of alcohol during a sham taste preference task ([6], Experiment 3).

## 5. Conclusions

Our findings make a contribution towards the currently very limited evidence base exploring the effects of RDMs. This study also represents, to our knowledge, the first explicit assessment of RDMs amongst a group of underage drinkers. The findings outlined here are not conclusive, but do suggest that factors such as active or passive engagement may have differential effects on different outcomes related to alcohol use cognitions. Further research needs to explore and verify these findings, and we would also suggest a need for more research exploring the contents of RDMs per se. Given the paucity of research in this area (see [3]) and the potential iatrogenic effects observed in this study when young people were exposed to RDMs, we would caution the use of such interventions amongst underage drinking groups unless and until a more robust evidence base is developed to ensure undesirable effects are not inadvertently provoked.

## Figures and Tables

**Table 1:** Pearson correlations between AUDIT-C scores and baseline measures of attitude and intention.

Variable	Attitude towards…		Intention to…		Beliefs about Frequency of…	
	1. Drinking Alcohol	2. Drunkenness	3. Drink Alcohol	4. Get Drunk	5. Drinking at 18	6. Drunkenness at 18
AUDIT-C	0.52 *	0.61 *	0.81 *	0.77 *	0.75 *	0.78 *
1		0.60 *	0.52 *	0.44 *	0.60 *	0.54 *
2			0.57 *	0.53 *	0.61 *	0.67 *
3				0.89 *	0.68 *	0.71 *
4					0.58 *	0.62 *
5						0.80 *

* *p* < 0.001.

**Table 2:** Mean responses to each dependent variable as a function of poster type, exposure condition, and time (pre- and post-intervention). Standard deviations shown in parentheses. *n* = 94.

Exposure Condition	Poster Type			
	Responsible Drinking		General Wellbeing	
	Passive	Active	Passive	Active
Time 1				
Attitude to drinking	4.0 (1.5)	4.0 (1.3)	4.3 (1.1)	4.1 (1.6)
Attitude to drunkenness	5.2 (1.7)	4.4 (1.5)	5.5 (1.4)	5.4 (1.6)
Intention to drink at weekend	3.0 (2.5)	3.5 (2.3)	2.6 (1.8)	3.6 (2.4)
Intention to get drunk at weekend	2.6 (2.6)	2.7 (2.1)	2.2 (1.9)	3.3 (2.3)
Drinking at 18	3.2 (1.2)	2.9 (1.2)	3.0 (1.0)	3.0 (1.2)
Drunkenness at 18	4.0 (1.1)	3.4 (1.2)	3.6 (1.0)	3.5 (1.0)
Time 2				
Attitude to drinking	3.9 (1.3)	3.7 (1.2)	4.4 (1.0)	4.6 (1.3)
Attitude to drunkenness	4.6 (1.6)	4.7 (1.5)	5.6 (1.2)	5.2 (1.6)
Intention to drink at weekend	3.3 (2.5)	4.2 (2.3)	2.8 (1.7)	3.2 (1.9)
Intention to get drunk at weekend	2.5 (2.3)	3.1 (2.3)	2.0 (1.8)	2.6 (1.7)
Drinking at 18	3.1 (1.2)	3.0 (1.2)	3.0 (1.0)	2.9 (1.1)
Drunkenness at 18	3.5 (1.3)	3.5 (1.1)	3.8 (1.0)	3.6 (1.1)

**Table 3:** MANCOVA showing multivariate and univariate effects of the independent variables on post-intervention changes in attitudes towards drinking alcohol and getting drunk. *n* = 94.

Variable	Multivariate Effects ^†^				Univariate Effects				
	*F*	*df*	*p*	*η_p_^2^*	Dependent Variable	*F*	*df*	*p*	*η_p_^2^*
Gender	0.39	6, 81	0.89	0.03	Att. Drinking	2.60	1, 86	0.11	0.03
					Att. Drunkenness	0.04	1, 86	0.54	<0.01
					Int. Drink Wknd.	1.49	1, 86	0.23	0.02
					Int. Get Drunk Wknd.	3.70	1, 86	0.06	0.04
					Drinking at 18	0.58	1, 86	0.45	0.01
					Drunkenness at 18	0.04	1, 86	0.84	<0.001
Location	1.03	6, 81	0.41	0.07	Att. Drinking	0.54	1, 86	0.47	0.01
					Att. Drunkenness	0.19	1, 86	0.66	<0.01
					Int. Drink Wknd.	0.12	1, 86	0.73	<0.001
					Int. Get Drunk Wknd.	0.02	1, 86	0.90	<0.001
					Drinking at 18	0.20	1, 86	0.65	<0.01
					Drunkenness at 18	0.68	1, 86	0.41	0.01
AUDIT-C	2.53	6, 81	0.03 *	0.16	Att. Drinking	2.23	1, 86	0.14	0.03
					Att. Drunkenness	9.28	1, 86	<0.01 **	0.10
					Int. Drink Wknd.	3.85	1, 86	0.053	0.04
					Int. Get Drunk Wknd.	5.67	1, 86	0.02	0.06
					Drinking at 18	0.30	1, 86	0.59	<0.01
					Drunkenness at 18	0.01	1, 86	0.92	<0.001
Poster Type	1.89	6, 81	0.09	0.12	Att. Drinking	4.92	1, 86	0.03 *	0.05
					Att. Drunkenness	0.09	1, 86	0.76	<0.001
					Int. Drink Wknd.	3.40	1, 86	0.07	0.04
					Int. Get Drunk Wknd.	2.63	1, 86	0.11	0.03
					Drinking at 18	0.57	1, 86	0.45	0.01
					Drunkenness at 18	3.51	1, 86	0.06	0.04
Exposure Condition	0.58	6, 81	0.74	0.04	Att. Drinking	0.12	1, 86	0.73	<0.001
					Att. Drunkenness	0.57	1, 86	0.45	0.01
					Int. Drink Wknd.	0.04	1, 86	0.84	<0.001
					Int. Get Drunk Wknd.	0.11	1, 86	0.74	<0.001
					Drinking at 18	0.04	1, 86	0.84	<0.001
					Drunkenness at 18	3.49	1, 86	0.07	0.04
Poster × Exposure	3.15	6, 81	0.01 **	0.19	Att. Drinking	1.44	1, 86	0.23	0.02
					Att. Drunkenness	8.70	1, 86	<0.01 **	0.09
					Int. Drink Wknd.	1.81	1, 86	0.18	0.02
					Int. Get Drunk Wknd.	1.58	1, 86	0.21	0.02
					Drinking at 18	0.60	1, 86	0.44	0.01
					Drunkenness at 18	8.76	1, 86	<0.01 **	0.09

^†^ Pillai’s trace, * *p* <0.05, ** *p* < 0.01.
